# The association between Mediterranean Diet Score and glucokinase regulatory protein gene variation on the markers of cardiometabolic risk: an analysis in the European Prospective Investigation into Cancer (EPIC)-Norfolk study

**DOI:** 10.1017/S0007114514000580

**Published:** 2014-05-07

**Authors:** Mercedes Sotos-Prieto, Robert Luben, Kay-Tee Khaw, Nicholas J. Wareham, Nita G. Forouhi

**Affiliations:** 1 Genetic and Molecular Epidemiology Unit, Department of Preventive Medicine and Public Health, School of Medicine, University of Valencia, Valencia, Spain. CIBER Fisiopatología de la Obesidad y Nutrición, Valencia, Spain; 2 Department of Epidemiology, Atherothrombosis and Cardiovascular Imaging, Centro Nacional de Investigaciones Cardiovasculares (CNIC), 28029Madrid, Spain; 3 MRC Epidemiology Unit, University of Cambridge School of Clinical Medicine, Institute of Metabolic Science, Cambridge Biomedical Campus, CambridgeCB2 0QQ, UK; 4 Department of Public Health and Primary Care, University of Cambridge, Cambridge, UK

**Keywords:** Mediterranean diet, Glucokinase regulatory protein, Cardiometabolic risk, Apolipoproteins, Lipids

## Abstract

Consumption of a Mediterranean diet (MD) and genetic variation in the glucokinase regulatory protein (*GCKR*) gene have been reported to be associated with TAG and glucose metabolism. It is uncertain whether there is any interaction between these factors. Therefore, the aims of the present study were to test the association of adherence to a MD and rs780094 (G>A) SNP in the *GCKR* gene with the markers of cardiometabolic risk, and to investigate the interaction between genetic variation and MD adherence. We studied 20 986 individuals from the European Prospective Investigation into Cancer (EPIC)-Norfolk study. The relative Mediterranean Diet Score (rMED: range 0–18) was used to assess MD adherence. Linear regression was used to estimate the association between the rMED, genotype and cardiometabolic continuous traits, adjusting for potential confounders. In adjusted analyses, we observed independent associations of MD adherence and genotype with cardiometabolic risk, with the highest risk group (AA genotype; lowest rMED) having higher concentrations of TAG, total cholesterol and apoB (12·5, 2·3 and 3·1 %, respectively) *v.* those at the lowest risk (GG genotype; highest rMED). However, the associations of MD adherence with metabolic markers did not differ by genotype, with no significant gene–diet interactions for lipids or for glycated Hb. In conclusion, we found independent associations of the rMED and of the *GCKR* genotype with cardiometabolic profile, but found no evidence of interaction between them.

The Mediterranean diet (MD) pattern has been associated with reduced CHD, cancer and overall mortality^(^ ^1^ ^)^. The MD is characterised by a relatively greater proportion of quantity and diversity of plant-derived foods (whole-grain cereals, raw and cooked vegetables, fresh and dried fruits, legumes and nuts), fish, a relatively moderate intake of meat and dairy products, with olive oil as the added fat, and a moderate intake of wine during meals^(^ ^1^ ^)^. Although different levels of adherence to the MD have been shown by country (with lower adherence in Northern Europe, medium in Central Europe and higher in Southern Europe among the countries included in the European Prospective Investigation into Cancer (EPIC)-InterAct study^(^ ^2^ ^)^), similar associations with CVD were observed across populations in different regions^(^ ^3^ ^)^. Despite some variation in findings^(^ ^4^ ^–^ ^7^ ^)^, the overall evidence from epidemiological and interventional studies suggests a beneficial effect of adherence to the MD on continuous metabolic traits of cardiovascular risk such as lipids and glycaemia^(^ ^4^ ^,^ ^8^ ^–^ ^11^ ^)^.

Recently, the potential importance of apolipoproteins in the prediction of cardiovascular risk has been appraised^(^ ^12^ ^,^ ^13^ ^)^, and an interventional study has recently reported an improvement in apolipoprotein profile (higher apoA-1, and lower apoB and apoB:A-1 ratio) among individuals in the MD intervention group compared with a control diet-supplemented group^(^ ^14^ ^)^. In addition to the potential beneficial effects of diet, it is now also acknowledged that genetic factors may influence the regulation of plasma lipids and glucose metabolism^(^ ^15^ ^,^ ^16^ ^)^, and that gene–diet interactions may help explain the variation in metabolic risk^(^ ^15^ ^)^. Genome-wide association studies have identified a number of SNP that may be associated with these traits^(^ ^16^ ^,^ ^17^ ^)^.

The rs780094 (G>A) SNP in the glucokinase regulatory protein (*GCKR*) gene has been associated with higher TAG concentrations^(^ ^16^ ^–^ ^20^ ^)^. Conversely, the A allele has been associated with lower insulin levels, lower glucose levels and lower diabetes prevalence^(^ ^17^ ^,^ ^18^ ^,^ ^21^ ^,^ ^22^ ^)^. In a high cardiovascular risk Spanish population (PREDIMED-Valencia (PREvencion con DIeta MEDiterranea-Valencia) study), we have recently reported that a MD modulated the association of *GCKR* gene variation on TAG concentrations^(^ ^23^ ^)^. However, it is currently unknown whether such effects may apply in the general British population, and whether they apply to other lipids, apolipoproteins and glycaemic markers, and whether there is an interaction between genetic and dietary factors on these metabolic parameters.

Therefore, the objectives of the present study were (1) to assess the association between adherence to the MD and continuous metabolic traits related to lipids and glucose metabolism in a large population-based study, (2) to investigate the association of *GCKR* rs780094 (G>A) with TAG concentrations and other metabolic markers and (3) to examine the joint effects and interaction of MD adherence and genetic effects on the metabolic markers of lipids and glycaemia.

## Subjects and methods

### Study participants and design

The EPIC-Norfolk Study recruited 25 639 men and women, aged 40–79 years at baseline (1993–7), who were resident in and around Norwich, England. The present study has been described in detail previously^(^ ^24^ ^)^, and it was conducted according to the guidelines laid down in the Declaration of Helsinki, and all procedures involving human participants were approved by the Norfolk District Health Authority Ethics Committee. Written informed consent was obtained from all participants. Since the baseline health-check visit, there were three follow-up assessments including two postal questionnaires and a repeat health-check visit, but this cross-sectional analysis is based on the baseline visit. Health and lifestyle information was collected using a baseline questionnaire, which asked about the participants' personal and family health, demography, lifestyle (including diet and physical activity) and social status (education and occupation). A standardised health check was performed by trained nurses, including measurement of height (cm), weight (kg) and waist circumference (WC; cm) as described previously^(^ ^24^ ^)^. Non-fasting blood samples were collected. A detailed description of the storage method has been described previously^(^ ^25^ ^)^. Concentrations of serum lipids, total cholesterol (TC), HDL-cholesterol (HDL-C) and TAG concentrations were measured on an RA-1000 (Bayer Diagnostics). LDL-cholesterol (LDL-C) was calculated using the Friedewald formula^(^ ^26^ ^)^; when serum TAG concentration exceeded 4·0 mmol/l, LDL-C concentration was not calculated. Concentrations of serum apoA-1 and apoB were measured using an Olympus AU640 Analyser (Olympus UK Limited). Measurement of glycated Hb (HbA1c) was added halfway through the baseline visit in 1995, and was available in approximately half of the cohort (*n* 10 780, with *n* 10 746 with HbA1c levels < 6·5 %). The level of HbA1c was measured with HPLC on a Bio-Rad Diamat (Bio-Rad).

We excluded the participants with missing baseline FFQ data or with ten missing FFQ lines as well as those with unavailable information on lipid measures. Thus, 20 986 participants from the baseline visit were eligible for inclusion in the present analysis.

### Dietary assessment and Mediterranean Diet Score

Participants completed a validated 130-item semi-quantitative FFQ about their habitual diet in the past year^(^ ^27^ ^)^. For all food items, respondents were asked to report the frequency of consumption on a 9-point scale for a ‘medium serving’ from ‘never or once per month’ to ‘more than six times per day’. Amounts of energy and individual nutrient intake were calculated from the frequency and amount (medium serving size) of each food reported in the FFQ and converted into g/d by using in-house software, the Compositional Analyses from Frequency Estimates (CAFE) program^(^ ^28^ ^)^. The EPIC FFQ was validated against 16 d weighed food records (*n* 127)^(^ ^29^ ^)^.

Adherence to the MD was assessed by using the relative Mediterranean Diet Score (rMED)^(^ ^30^ ^,^ ^31^ ^)^, which is a variation of the original MD Score^(^ ^1^ ^)^. This variation consists mainly in the score criterion and in the olive oil component. Similarly to the MD Score, the rMED included nine nutritional components that are characteristic of the MD and included some presumed ‘beneficial’ components (vegetables, legumes, fruit and nuts, cereals, fish and seafood, olive oil (including olive oil consumption instead of the ratio of monounsaturated fat:saturated fat as in the original MD) and moderate alcohol consumption) and other presumed ‘detrimental’ components (meat and meat products and dairy products), as described previously^(^ ^2^ ^,^ ^30^ ^,^ ^31^ ^)^. While the original MD Score criteria assigned 1 point for intakes above the median among the study participants and 0 point for intakes below the median, with reversed scoring for meat and dairy intakes (score range 0–9), in the rMED, each component (apart from alcohol consumption) was calculated as a function of energy density (as g/4184 kJ (1000 kcal)), and was then divided into tertiles of intakes. We assigned a value of 0, 1 or 2 to the first, second and third tertiles, respectively, of the intakes of vegetables, legumes, fruit and nuts, cereals, and fish and seafood and positively scoring higher intakes for the beneficial components. The scoring was reversed for the two presumed detrimental components (meat and meat products and dairy products) by assigning a higher score for lower intakes. The scoring for olive oil was modified for the rMED^(^ ^31^ ^)^ because of the relatively large number of non-consumers. Therefore, 0 was assigned to non-consumers, 1 was assigned to participants with an intake below the median olive oil consumption (calculated only within olive oil consumers) and 2 was assigned to participants whose intake was equal to or above this median. For alcohol, a value of 2 was given to men with moderate alcohol consumption (intakes from 10 to 50 g/d), and a value of 0 was assigned otherwise, whereas for women, the corresponding cut-off points were 5 and 25 g/d. Therefore, the rMED ranged from 0 (indicating the lowest adherence to the MD) to 18 (indicating the highest adherence to the MD), which is different from the original MD Score that ranged between 0 and 9. Finally, the rMED was further classified into categories to reflect low (0–6 points), medium (7–10 points) or high (11–18 points) adherence to the MD on the basis of previously published cut-off points^(^ ^30^ ^)^.

### Determination of genotypes

DNA for genotyping was extracted from EDTA whole-blood aliquots collected at the first and second health checks using phenol–chloroform extraction. The call rate was 99·5 %. There were 19 800 participants with available data on genotype (rs780094). We also included data on rs1260326 SNP (in high linkage disequilibrium with the rs780094 SNP; *r* ^2^ 0·94) in a subset (*n* 7273), to replicate findings. Genotype distributions did not deviate from Hardy–Weinberg expectations (*P*= 0·14, rs780094 and *P*= 0·80, rs1260326).

### Statistical analysis

Continuous variables were assessed for normality of distribution, and skewed variables were normalised by log_10_ transformation. Data are presented as means and standard deviations or geometric means and 95 % CI for continuous variables, and frequencies and percentages for categorical variables. Baseline characteristics were described by rMED categories (low, medium and high). Differences in means among the groups were compared by using a one-factor ANOVA test, and comparisons of frequencies were conducted using the χ^2^ test. We fitted multiple linear regression models for the association between the rMED categories and continuous metabolic traits (TAG, TC, LDL-C, HDL-C, apoA-1, apoB and HbA1c) in the following way: model 1 adjusted for sex, age (in years, continuous). Model 2 additionally adjusted for potential confounders including physical activity (self-reported) that was derived into a four-scale index by combining levels of occupational and recreational physical activity^(^ ^32^ ^)^ (inactive, moderately inactive, moderately active and active), smoking status (current smoker, former smoker and never smoker), total energy intake (EI, in kJ, continuous), lipid-lowering medication, social class (professional, managerial and technical, skilled non-manual, partly skilled and unskilled), and educational level (low education, O-level or equivalent to secondary school, A-level or equivalent including technical school, university degree or equivalent, including a higher vocational qualification). Model 3 additionally adjusted for BMI (continuous) and WC (continuous). We also examined the effects of per two-point increase in the rMED (as a continuous variable). The association between adherence to the MD and hypertriacylglycerolaemia status (defined according to the cut-off points ( ≥ 1·7 mmol TAG) was determined using multivariable-adjusted logistic regression models. We performed sensitivity analyses by excluding participants with chronic prevalent disease (heart disease, stroke, diabetes mellitus and/or cancer) and excluding misreporters of energy. Misreporting of EI was estimated by using the ratio of reported EI:predicted BMR (EI:BMR). Participants were classified as under-reporters (EI:BMR < 1·14), plausible reporters (EI:BMR = 1·14–2·1) or over-reporters (EI:BMR>2·1) of EI by using cut-off points proposed by Goldberg^(^ ^33^ ^)^.

Among the 19 800 individuals with available data, we analysed the rs780094 SNP using a co-dominant mode of inheritance as in previous studies^(^ ^19^ ^,^ ^23^ ^,^ ^34^ ^)^. In multiple linear regression analysis, we adjusted for relevant covariates, with model 1 including age (continuous) and sex, and model 2 additionally including BMI and WC (both continuous) and use of lipid-lowering medication (no or yes). Multivariable logistic regression was used to estimate the OR of hypertriacylglycerolaemia associated with the polymorphism.

Joint effects of genotype (GG, GA and AA) and rMED (low, medium and high adherence to the MD) on continuous metabolic traits were examined using ANCOVA with nine possible combinations (high adherence+GG, high adherence+GA, high adherence+AA, medium adherence+GG, medium adherence+GA, medium adherence+AA, low adherence+GG, low adherence+GA and low adherence+AA).

To examine the gene × MD adherence interaction on the levels of cardiometabolic markers (TAG, TC, apoB and HbA1c), we used multivariable linear regression models including main effects and interaction terms.

Statistical analyses were conducted using SPSS version 15.0 for Windows (SPSS, Inc.). Statistical significance was set at the 0·05 level, and all tests were two-tailed. In addition, we also applied the Bonferroni method for multiple comparisons, and set a lower statistical significance accounting for the number of tests of significance^(^ ^35^ ^)^.

## Results

Among the 20 986 individuals, 33 % (*n* 6924), 51 % (*n* 10 627), and 16 % (*n* 3435) were in the low, medium and high MD adherence categories (rMED).

Overall, a higher adherence to the MD was observed among women, individuals with a lower BMI and WC, older, physically active people, and individuals with a higher level of education (Table 1).

**Table 1:** Baseline characteristics according to the levels of adherence to Mediterranean dietary patterns (relative Mediterranean Diet Score (rMED) categories) in the European Prospective Investigation into Cancer (EPIC)-Norfolk study participants (Mean values and standard deviations; percentages; median values and interquartile ranges (IQR))

			rMED categories*	*P*	
	Total†	Low†	Medium†	High†
	Mean	sd	Mean	sd	Mean	sd	Mean	sd
Age (years)	59·3	9·3	59·0	9·4	59·4	9·2	59·3	9·3	0·018
Men (%)	45·3	47·2	44·6	43·6	< 0·001				
BMI (kg/m^2^)	26·3	3·8	26·2	3·8	26·4	3·8	26·1	3·8	0·005
WC (cm)	88·1	12·3	89·3	12·1	87·9	12·3	86·4	12·3	< 0·001
Social class (%)									
Uncoded	0·3	0·4	0·3	0·2	
Professional	7·0	6·3	7·0	8·7	< 0·001
Managerial and technical	36·8	33·5	37·4	41·5	
Skilled non-manual	16·6	16·5	16·5	16·8	
Skilled manual	22·9	24·9	22·6	19·5	
Partly skilled	13·1	14·4	13·0	10·9	
Unskilled	3·3	3·4	3·2	2·4	
Educational level (%)									
Low	36·6	39·2	36·5	31·5	< 0·001
O-level or equivalent	10·2	9·9	10·5	9·8	
A-level or equivalent	40·3	39·3	40·2	42·9	
University degree or equivalent	12·9	11·7	12·8	15·8	
PA (%)									
Inactive	30·1	31·9	29·8	27·1	< 0·001
Moderately inactive	28·8	27·2	29·3	30·1	
Moderately active	22·8	22·8	22·9	22·3	
Active	18·4	18·1	17·9	20·5	
Smoking (%)									
Never	11·4	14·4	10·6	7·6	< 0·001
Former	42·4	41·0	42·7	44·4	
Current	44·6	46·8	46·1	46·3	
Lipid medication, yes (%)	1·5	0·8	1·7	2·5	< 0·001
Dietary components of the rMED (g/d)									
Overall rMED									< 0·001
Median	7·8	4·8	8·4	11·9	
IQR	2·7	1·2	1·1	1·1	
Vegetables									< 0·001
Median	227·1	175·6	241·5	306·7	
IQR	129·8	111·3	144·3	151·9	
Fruit and nuts									< 0·001
Median	163·4	151·8	228·2	296·7	
IQR	54·2	152·6	195·5	207·9	
Legumes									< 0·001
Median	57·2	38·9	56·6	70·9	
IQR	57·2	38·4	41·3	44·9	
Fish									< 0·001
Median	34·6	27·2	34·6	43·4	
IQR	28·3	22·3	26·1	35·0	
Cereals									< 0·001
Median	262·8	234·6	250·1	271·6	
IQR	120·6	113·9	125·3	133·4	
Olive oil									0·625
Median	0·98	0·8	0·9	1·0	
IQR	0·98	1·1	0·9	1·0	
Alcohol									< 0·001
Median	4·7	2·3	4·7	7·8	
IQR	10·1	7·3	10·7	10·7	
Meat and meat products									< 0·001
Median	107·0	114·6	94·4	72·2	
IQR	78·2	66·5	60·9	52·2	
Dairy products									< 0·001
Median	90·2	96·5	80·1	63·0	
IQR	47·8	81·8	77·4	65·0	

WC, waist circumference, PA, physical activity.

* Low adherence to the Mediterranean diet (MD; rMED 0–6); medium adherence to the MD (rMED 7–10); high adherence to the MD (rMED 11–18).

† Total: *n* 20 986; low: *n* 6924 (33 %); medium: *n* 10 627 (51 %); high: *n* 3435 (16 %).

### Association between Mediterranean diet adherence and metabolic markers

Table 2 shows that TAG concentrations and the apoB:apoA-1 ratio in the medium and high categories of the rMED were significantly lower, while HDL-C and apoA-1 concentrations were higher compared with the concentrations in the lowest rMED category. Further mutual adjustment for other lipids subtypes did not alter the findings. HbA1c concentration was lower with an increasing rMED. After Bonferroni correction for multiple comparisons, each of the concentrations of TAG, HDL-C and HbA1c remained significantly associated with the rMED.

**Table 2:** Association between adherence to the Mediterranean diet (MD) according to the relative Mediterranean Diet Score (rMED) categories and metabolic markers in the European Prospective Investigation into Cancer-Norfolk study participants (Mean or geometric mean values and 95 % confidence intervals)

	rMED categories*	
	Low†	Medium†	High†	
	Mean	95 % CI	Mean	95 % CI	Mean	95 % CI	*P* ‡
TAG (mmol/l)§							
Crude	1·60	1·58, 1·62	1·53	1·52, 1·55	1·47	1·45, 1·50	< 0·001
Model 1∥	1·58	1·56, 1·60	1·54	1·52, 1·55	1·50	1·48, 1·52	< 0·001
Model 2¶	1·57	1·55, 1·58	1·54	1·52, 1·50	1·51	1·49, 1·53	0·001
Model 3**	1·57	1·55, 1·58	1·53	1·52, 1·55	1·52	1·50, 1·54	0·001
TC (mmol/l)§							
Crude	6·04	6·01, 6·07	6·08	6·05, 6·10	6·10	6·05, 6·14	0·037
Model 1	6·07	6·05, 6·10	6·07	6·04, 6·08	6·07	6·03, 6·11	0·828
Model 2	6·08	6·05, 6·10	6·05	6·04, 6·08	6·05	6·01, 6·10	0·468
Model 3	6·08	6·05, 6·11	6·05	6·04, 6·08	6·05	6·01, 6·10	0·356
LDL-C (mmol/l)§							
Crude	3·84	3·81, 3·86	3·85	3·84, 3·87	3·83	3·79, 3·86	0·443
Model 1	3·85	3·83, 3·87	3·85	3·78, 3·85	3·82	3·78, 3·85	0·312
Model 2	3·85	3·83, 3·88	3·84	3·82, 3·86	3·81	3·77, 3·85	0·143
Model 3	3·85	3·83, 3·88	3·84	3·82, 3·85	3·81	3·77, 3·85	0·119
HDL-C (mmol/l)§							
Crude	1·31	1·30, 1·31	1·36	1·35, 1·36	1·42	1·41, 1·42	< 0·001
Model 1	1·33	1·32, 1·34	1·35	1·34, 1·36	1·39	1·37, 1·40	< 0·001
Model 2	1·34	1·33, 1·35	1·35	1·34, 1·35	1·38	1·37, 1·39	< 0·001
Model 3	1·34	1·33, 1·35	1·35	1·34, 1·36	1·38	1·36, 1·39	< 0·001
ApoA-1 (mmol/l)§							
Crude	1·52	1·51, 1·53	1·56	1·55, 1·56	1·60	1·58, 1·61	< 0·001
Model 1	1·54	1·54, 1·55	1·55	1·55, 1·56	1·57	1·56, 1·58	0·001
Model 2	1·54	1·54, 1·55	1·55	1·54, 1·56	1·57	1·56, 1·58	0·008
Model 3	1·54	1·56, 1·55	1·55	1·54, 1·56	1·57	1·56, 1·58	0·007
ApoB (mmol/l)§							
Crude	0·97	0·96, 0·98	0·98	0·97, 0·98	0·97	0·96, 0·98	0·319
Model 1	0·97	0·97, 0·98	0·97	0·97, 0·98	0·97	0·96, 0·98	0·654
Model 2	0·97	0·97, 0·98	0·97	0·97, 0·98	0·97	0·96, 0·98	0·462
Model 3	0·97	0·97, 0·98	0·97	0·97, 0·98	0·97	0·96, 0·98	0·505
ApoB:apoA-1							
Crude	0·65	0·65, 0·66	0·64	0·64, 0·65	0·63	0·62, 0·63	< 0·001
Model 1	0·64	0·64, 0·65	0·65	0·64, 0·65	0·63	0·63, 0·64	0·018
Model 2	0·65	0·64, 0·65	0·65	0·64, 0·65	0·63	0·63, 0·64	0·015
Model 3	0·65	0·64, 0·65	0·64	0·64, 0·65	0·63	0·63, 0·64	0·027
HbA1c (%)§ ††							
Crude	5·250	5·22, 5·27	5·19	5·17, 5·20	5·16	5·13, 5·18	< 0·001
Model 1	5·25	5·23, 5·27	5·19	5·17, 5·20	5·16	5·13, 5·18	< 0·001
Model 2	5·24	5·22, 5·26	5·19	5·17, 5·20	5·17	5·14, 5·20	< 0·001
Model 3	5·24	5·22, 5·26	5·19	5·17, 5·20	5·17	5·14, 5·20	< 0·001

TC, total cholesterol; HDL-C, HDL cholesterol; LDL-C, LDL cholesterol; HbA1c, glycated Hb.

* Low adherence to the MD (rMED 0–6); medium adherence to the MD (rMED 7–10); high adherence to the MD (rMED 11–18).

† Low adherence: *n* 6924 (33 %); medium: *n* 10 627 (51 %); high: *n* 3435 (16 %).

‡ *P* value is shown for the one-factor ANOVA test. A lower *P* value of 0·00 625 was applied after using the Bonferroni correction method for multiple comparisons.

§ All the variables are log-transformed.

∥ Model 1: adjusted for age and sex.

¶ Model 2: model 1+social class, educational level, physical activity, smoking status, energy intake and lipid-lowering medication.

** Model 3: model 2+BMI and waist circumference.

†† HbA1c *n* 10 746, with HbA1c levels < 6·5 %.

Each two-point increment in the rMED was significantly inversely associated with TAG (β − 0·005, 95 % CI − 0·007, − 0·002), LDL-C (β − 0·001, 95 % CI − 0·003, 0·000), apoB:apoA-1 ratio (β − 0·002, 95 % CI − 0·005, 0·000) and HbA1c (β − 0·001, 95 % CI − 0·002, 0·000), while it was directly associated with HDL-C (β 0·004, 95 % CI 0·002, 0·005) and apoA-1 (β 0·006, 95 % CI 0·002, 0·009) (β coefficients for log-transformed variables). The rMED was inversely associated with hypertriacylglycerolaemia (TAG>1·7 mmol/l), with OR 0·89, 95 % CI 0·81, 0·98; model 3. Sensitivity analyses did not alter the main findings.

### Association between glucokinase regulatory protein SNP and metabolic markers

Baseline characteristics according to the *GCKR* rs780094 SNP showed that no significant differences were observed in relation to age, sex, BMI, WC, social class and physical activity. Significant differences were found for educational level and lipid medication (see online Supplementary Table S1). The AA genotype (frequency 15·6 %) was associated with 0·16 mmol/l higher TAG concentrations compared with GG individuals in adjusted analyses (Table 3). The AA genotype was also significantly positively associated with TC and apoB, but no significant differences were found for other lipid parameters (HDL-C and apoA-1) by genotype (Table 3). We found a tendency towards lower HbA1c concentration for the A allele, but this was not statistically significant. Application of the Bonferroni method for multiple comparisons did not alter these findings. Both GA and AA genotypes *v.* GG genotype were associated with a greater risk of hypertriacylglycerolaemia in adjusted analyses (GA: OR 1·23, 95 % CI 1·15, 1·33; AA: OR 1·47, 95 % CI 1·33, 1·62).

**Table 3:** Association between genotypes in the glucokinase regulatory protein (*GCKR*) gene (rs780094 (G>A)) and metabolic markers in the European Prospective Investigation into Cancer (EPIC)-Norfolk study participants (Mean or geometric mean values and 95 % confidence intervals)

	rs780094 (G>A)	
	GG*	GA*	AA*	
	Mean	95 % CI	Mean	95 % CI	Mean	95 % CI	*P* †
TAG (mmol/l)‡							
Crude	1·50	1·48,1·52	1·58	1·57,1·60	1·66	1·63,1·69	< 0·001
Model 1§	1·51	1·50,1·52	1·58	1·55,1·60	1·66	1·63,1·69	< 0·001
Model 2∥	1·50	1·48,1·52	1·58	1·57,1·60	1·66	1·64,1·69	< 0·001
TC (mmol/l)‡							
Crude	6·03	6·00,6·05	6·07	6·04,6·08	6·14	6·09,6·18	< 0·001
Model 1	6·03	3·00,6·05	6·07	6·04,6·08	6·14	6·09,6·18	< 0·001
Model 2	6·03	3·00, 6·05	6·07	6·04, 6·08	6·14	6·09,6·18	< 0·001
LDL-C (mmol/l)‡							
Crude	3·84	3·81,3·86	3·83	3·81,3·85	3·90	3·84,3·92	0·173
Model 1	3·85	3·82,3·86	3·83	3·81,3·85	3·90	3·84,3·92	0·179
Model 2	3·84	3·82,3·86	3·83	3·81,3·85	3·87	3·84,3·92	0·137
HDL-C (mmol/l)‡							
Crude	1·34	1·33,1·35	1·34	1·33,1·35	1·33	1·32,1·35	0·798
Model 1	1·33	1·32,1·34	1·34	1·33,1·35	1·33	1·32,1·35	0·612
Model 2	1·34	1·33,1·34	1·34	1·33,1·35	1·33	1·32,1·34	0·574
ApoA-1 (mmol/l)‡							
Crude	1·54	1·53,1·54	1·54	1·54,1·55	1·55	1·53,1·56	0·231
Model 1	1·54	1·53,1·55	1·55	1·54,1·56	1·55	1·54,1·57	0·083
Model 2	1·54	1·53,1·55	1·55	1·54,1·55	1·55	1·54,1·56	0·104
ApoB (mmol/l)‡							
Crude	0·96	0·96,0·97	0·97	0·97,0·98	0·99	0·98,1·00	< 0·001
Model 1	0·97	0·96,0·97	0·97	0·97,0·98	0·99	0·98,1·00	0·001
Model 2	0·97	0·96, 0·97	0·97	0·97,0·98	0·99	0·98,1·00	< 0·001
ApoB:apoA-1							
Crude	0·64	0·64,0·65	0·65	0·64,0·65	0·66	0·65,0·66	0·018
Model 1	0·65	0·64,0·65	0·64	0·64, 0·65	0·65	0·65, 0·66	0·092
Model 2	0·65	0·64, 0·65	0·64	0·64, 0·65	0·66	0·65, 0·66	0·050
HbA1c (%)‡ ¶							
Crude	5·28	5·26, 5·31	5·26	5·24, 5·28	5·24	5·20, 5·28	0·232
Model 1	5·28	5·26,5·31	5·26	5·24, 5·28	5·24	5·20, 5·28	0·143
Model 2	5·28	5·26, 5·31	5·26	5·24, 5·28	5·20	5·21, 5·28	0·271

TC, total cholesterol; HDL-C, HDL cholesterol; LDL-C, LDL cholesterol; HbA1c, glycated Hb.

* GG: *n* 7372 (37·2 %); GA: *n* 9337 (47·2 %), AA: *n* 3091 (15·6 %).

† *P* value is shown for the one-factor ANOVA test. A lower *P* value of 0·00 625 was applied after using the Bonferroni correction method for multiple comparisons.

‡ All the variables are log-transformed.

§ Model 1: adjusted for age and sex.

∥ Model 2: model 1+BMI+waist circumference+lipid-lowering medication.

¶ HbA1c *n* 10 746, with HbA1c levels < 6·5 %.

### Joint results of genotype and Mediterranean diet adherence on metabolic markers

Table 4 demonstrates the joint results of genotype and MD adherence on metabolic parameters. Participants with the highest risk (AA genotype and with the lowest rMED) had 12·5 % higher TAG concentrations (mean 1·68 (95 % CI 1·63, 1·73) mmol/l) than those who had the lowest risk (mean 1·47 (95 % CI 1·45, 1·50) mmol/l; GG genotype with the highest rMED; Table 4). Similar results were found for TC concentrations (2·3 % higher) and apoB (3·1 % higher), respectively (Table 4). For HbA1c, the converse was the case with those who were GG homozygous with the lowest rMED having 3·3 % higher HbA1c concentration (Table 4). However, the association of MD adherence and metabolic markers did not differ significantly by genotype, with no significant gene–diet interactions for lipids or for HbA1c (TAG, *P*= 0·770; TC, *P*= 0·761; apoB, *P*= 0·860; HbA1c, *P*= 0·481). These findings were unchanged with correction for multiple comparisons using the Bonferroni method.

**Table 4:** Combined association between the relative Mediterranean Diet Score (rMED; low, medium or high adherence to Mediterranean dietary patterns) and the genotype (rs780094 (G>A) SNP in the glucokinase regulatory protein (*GCKR*) gene) on lipid concentrations and glycated Hb (HbA1c) (Mean or geometric mean values and 95 % confidence intervals)

	GG*	GA*	AA*		
	Mean	95 % CI	Mean	95 % CI	Mean	95 % CI	*P* †	*P* for interaction
TAG (mmol/l)‡								
Low	1·53	1·51,1·57	1·63	1·60,1·66	1·68	1·63,1·73	< 0·001	0·770
Medium	1·49	1·47,1·52	1·57	1·54, 1·59	1·66	1·62, 1·69		
High	1·47	1·45,1·50	1·53	1·50, 1·57	1·64	1·57, 1·71		
*P* §	0·006		< 0·001		0·605			
TC (mmol/l)‡								
Low	6·02	5·97,6·07	6·08	6·04,6·12	6·18	6·11,6·25	0·002	0·761
Medium	6·02	5·97,6·07	6·05	6·01, 6·08	6·12	6·07,6·18		
High	6·04	5·97,6·11	6·07	6·01,6·14	6·11	6·00, 6·21		
*P* §	0·872		0·555		0·433			
ApoB (mmol/l)‡								
Low	0·96	0·95, 0·97	0·97	0·96, 0·98	1·00	0·98, 1·01	0·029	0·860
Medium	0·97	0·96, 0·98	0·97	0·96, 0·98	0·99	0·98, 1·0		
High	0·97	0·95,0·99	0·97	0·96,0·98	0·99	0·96, 1·0		
*P* §	0·737		0·947		0·747			
HbA1c (%)‡								
Low	5·33	5·29, 5·38	5·27	5·24, 5·32	5·28	5·22, 5·36	0·015	0·481
Medium	5·26	5·22, 5·30	5·26	5·24, 5·32	5·25	5·19, 5·30		
High	5·25	5·20, 5·31	5·21	5·16, 5·27	5·16	5·07, 5·26		
*P* §	0·083		0·047		0·161			

TC, total cholesterol.

* GG: *n* 7372 (37·2 %); GA: *n* 9337 (47·2 %), AA: *n* 3091 (15·6 %).

† *P* value for difference for the one-factor ANOVA test (model adjusted for age sex, BMI and lipid-lowering medication).

‡ All the variables are log-transformed.

§ *P* value for comparison between each genotype according to the rMED. A lower *P* value of 0·0125 was applied after using the Bonferroni correction method for multiple comparisons.

Similar results were found when we analysed the rs1260326 SNP (in high linkage disequilibrium with the rs780094 SNP, *r* ^2^ 0·94) in a small sample (*n* 7273; data not shown). Furthermore, we performed a subgroup analysis stratified by age ( < 59·1 years old *v.* >59·1 years old; cut-off points selected by median), physical activity (inactive or moderately inactive *v.* active or moderately active) and smoking status (smoker *v.* non-smoker) for the outcomes analysed in order to examine whether in any of these strata, a significant gene–diet interaction was obtained. We did not find any interaction, though there were differences by genotype in TAG levels in both strata for each of those three factors (data not shown).

## Discussion

The findings from the present large population-based study in the UK suggest that higher adherence to the MD is associated with important metabolic benefits for lipid and glycaemic parameters, while the AA genotype of the *GCKR* rs780094 SNP is associated with an adverse distribution of lipid parameters. We also report that the TAG-raising A allele of rs780094 is associated with a trend towards a HbA1c-lowering effect. While there were significant differences in metabolic parameter distribution when comparing those with the highest and lowest risks (i.e. those with highest genetic susceptibility and lowest MD adherence *v.* those with lowest genetic susceptibility and highest MD adherence), the benefits of greater MD adherence were not differential by genotype.

The present study extends previous findings that were demonstrated for TAG concentrations and genotype in a high-cardiovascular risk Spanish population of 945 older adults^(^ ^23^ ^)^, to several metabolic parameters in a general British population in the EPIC-Norfolk study. The results of the present study indicate that a high adherence to the MD as defined by the rMED is associated with an improved metabolic profile (lower TAG concentrations, higher HDL-C concentrations and lower HbA1c levels). We provide evidence that the rs789004 SNP in the *GCKR* gene is associated with TAG concentrations, confirming previous findings^(^ ^17^ ^,^ ^19^ ^,^ ^22^ ^,^ ^34^ ^,^ ^36^ ^)^, and additionally report that carriers of the minor A allele also have higher TC and apoB concentrations. We further demonstrate that there are no gene–diet interactions on metabolic parameters, but that greater adherence to the MD confers a more favourable metabolic profile irrespective of genetic susceptibility.

The results of the present study are in general agreement with other cross-sectional, prospective and interventional studies of the association between MD adherence and markers of cardiometabolic risk^(^ ^8^ ^,^ ^9^ ^,^ ^11^ ^,^ ^37^ ^–^ ^42^ ^)^. However, although some studies have found inverse associations between MD adherence and TC^(^ ^5^ ^,^ ^8^ ^)^, we did not find such an association for TC. A weak association was shown for LDL-C when we considered rMED as continuous (per two points of increase in the rMED). These findings could be attributable to the differences in the definition of rMED in different studies. We also found an inverse association between the rMED and HbA1c levels, a finding that is in line with our recent report of an inverse association between adherence to the MD and incident diabetes^(^ ^2^ ^)^.

Thus far, the association between MD adherence and apolipoproteins has not been widely studied. Notably, we found a direct association of MD adherence with apoA-1 concentration, but this was of borderline significance after Bonferroni correction for multiple comparisons, though it could be of potential clinical significance. This finding in a British general population is consistent with a randomised study in a high-cardiovascular risk Spanish population^(^ ^14^ ^)^. Our findings raise the possibility that MD adherence may be useful for achieving healthy apolipoprotein goals in the general British population, but require confirmation in future studies.

We confirmed the genetic association of the rare variant of the rs780094 SNP with higher TAG concentrations as described previously^(^ ^16^ ^–^ ^19^ ^,^ ^22^ ^)^. In the first study that identified this SNP in the *GCKR* gene as a target for TAG concentrations, this SNP was reported to explain 1 % of the residual variance in TAG levels^(^ ^17^ ^)^. In the present study, 0·5 % of the residual variance in TAG concentration is explained by this SNP, which is similar to that reported in Scandinavian samples (0·4–1·2 %)^(^ ^18^ ^)^ and higher than the variance reported in a French population (0·2 %)^(^ ^22^ ^)^. Past studies have found opposing associations of this SNP with TAG (elevated concentrations) and glucose (lowered concentrations)^(^ ^17^ ^,^ ^18^ ^,^ ^21^ ^,^ ^22^ ^,^ ^34^ ^)^. We have extended this finding by examining the association with HbA1c concentration, not previously reported in the literature, and we report that the TAG-raising A allele of rs780094 is associated with an HbA1c-lowering effect. A mutational mechanism for the reported association of this variant with raised TAG and lower glucose levels has been proposed, such that the *GCKR* T-allele Pro446Leu (C>T; rs1260326) has reduced regulation by physiological concentrations of phosphate esters fructose 6, resulting indirectly in increased glucokinase activity^(^ ^43^ ^)^. Altered glucokinase regulation in the liver is predicted to enhance glycolytic flux, promoting hepatic glucose metabolism and elevating concentrations of malonyl-CoA, a substrate for *de novo* lipogenesis, and thus relating to the reported association of this variant with raised TAG and lower glucose levels. Taken together, these data support an important role of GCKR in pathways regulating hepatic TAG as well as glucose metabolism in humans.

With respect to the study of genotype and other metabolic traits, we found an association between the rs780094 SNP and TC and apoB. Few studies have analysed other metabolic lipid or apolipoprotein traits in relation to this SNP^(^ ^18^ ^–^ ^20^ ^,^ ^36^ ^)^. Sparsø *et al.* ^(^ ^20^ ^)^ found a modest association between rs780094 and TC, and interestingly, in agreement with Chasman *et al.* ^(^ ^36^ ^)^, apoB concentrations are also higher in carriers of the risk allele in the present study.

Although no significant gene–diet interaction was found, this is the first study to examine how a SNP (rs780094) in the *GCKR* gene previously shown to be consistently associated with TAG concentrations is associated not only with TAG levels, but also with other cardiometabolic risk phenotypes (TC, apoB and HbA1c) according to the groups of adherence to the MD and genetic risk allele carriers.

The strengths of the present study include a large population-based sample size of approximately 20 000 individuals, and a well-phenotyped dataset that enabled us to account for a number of relevant plausible confounders and to estimate differences and interactions with a high degree of precision. A further strength is that we have extended previous findings from nutrigenetic studies that have focused on associations between single diet or nutrient components and endpoints to examine dietary patterns by assessing the rMED score of MD adherence. The analysis of dietary patterns may provide a more accurate depiction of peoples' eating habits and yield better models for generating public health recommendations regarding healthy eating^(^ ^44^ ^)^. Limitations of the present study include the cross-sectional design of the study that does not allow temporal relationships to be examined. The FFQ for dietary assessment is prone to measurement error, and its use at a single time point in the present study does not permit the examination of any changes in diet over time, notwithstanding, the EPIC-Norfolk FFQ is a validated instrument^(^ ^29^ ^)^. We had non-fasted samples in the present study, due to logistical issues relating to recruiting participants to a large epidemiological study. However, for glycaemia, we used HbA1c levels, where fasting is not required, and we do not believe that the absence of fasted lipid samples affects our overall findings and interpretation, in keeping with recent evidence^(^ ^12^ ^,^ ^45^ ^)^.

In conclusion, the present study provides evidence on the potential protective properties of the MD, evaluated as an *a priori*-defined score, on the markers of cardiometabolic risk, and confirms the association of genetic variation in *GCKR* with TAG concentrations, adding new information on its association with other lipids and apolipoproteins. The finding of both separate and joint associations of diet and genotype, but not an interaction between them, with the metabolic profile should be further investigated in other populations and in prospective studies.

## Supplementary material

To view supplementary material for this article, please visit http://dx.doi.org/10.1017/S0007114514000580
